# Draft Genome Sequence of *Methylosinus* sp. Strain 3S-1, an Isolate from Rice Root in a Low-Nitrogen Paddy Field

**DOI:** 10.1128/genomeA.00932-16

**Published:** 2016-09-01

**Authors:** Zhihua Bao, Ryo Shinoda, Kiwamu Minamisawa

**Affiliations:** Graduate School of Life Sciences, Tohoku University, Sendai, Japan

## Abstract

N_2_-fixing methanotrophs play an important role in the methane-nitrogen cycle in rice paddies. We report here the draft genome sequence of *Methylosinus* sp. strain 3S-1 isolated from rice root in a paddy field without N fertilizer input.

## GENOME ANNOUNCEMENT

Methane, a greenhouse gas, is emitted into the atmosphere through paddy rice plants (1). The methanotrophs in rice roots (2, 3) contribute to methane consumption (4, 5). Most methanotrophic bacteria possess nitrogen fixation genes and are able to fix N_2_ under laboratory conditions (6). A recent metaproteomic study (7) strongly suggested that type II methanotrophs, including *Methylosinus* spp., mediate both CH_4_ oxidation and N_2_ fixation in the root tissues of rice plants grown in a paddy field without nitrogen fertilization input. In addition, the *Methylosinus* genus was significantly abundant in rice root in the paddy field by metagenome analyses (8).

Rice roots (*Oryza sativa* L. cv. Nipponbare) grown in a low-N paddy field of Kashimadai Experimental Station (Tohoku University, Japan [38°27′37′′N and 141°5′33′′E]) were surface sterilized with 1% (wt/vol) NaOCl solution (August 2012). The root segments were placed on nitrate mineral salts (NMS) agar medium (9) and incubated at 30°C in chambers charged with 40% (vol/vol) CH_4_ in the air. Single-colony isolation on the plates was repeated several times in intervals of 2 to 4 weeks. The cells from the resultant single colonies were serially diluted from 10^-1^ to 10^-10^ in liquid NMS medium and then cultivated under 40% (vol/vol) CH_4_ in the air, which was repeated three or four times by using the highest dilutions. Among the resultant methanotrophs, we finally obtained strain S3-1 of *Methylosinus*.

The genomic DNA of *Methylosinus* sp. 3S-1 was sequenced by using paired-end sequencing with an Illumina MiSeq sequencer (New England BioLabs, Ipswich, MA, USA). Raw reads were trimmed and *de novo* assembled using the CLC Genomics Workbench version 8.5.1 (Qiagen, Valencia, CA, USA). The parameters for trimming were as follows: ambiguous limit, 2; quality limit, 0.05; number of 5′-terminal nucleotides, 20; number of 3′-terminal nucleotides, 5. The parameters for the *de novo* assembly were as follows: mapping mode, create simple contig sequences (fast); bubble size, 50; word size, 21; minimum contig length, 1,000 bp; perform scaffolding, no; autodetect paired distances, yes.

The draft genome of *Methylosinus* sp. 3S1 was assembled into 159 contigs, with an accumulated length of 4,762,464 bp (*N*_50_, 73,505 bp) and an average G+C content of 65.9%. The genome was annotated by the NCBI Prokaryotic Genome Annotation Pipeline (PGAP, version 3.3) (http://www.ncbi.nlm.nih.gov/genome/annotation_prok), and a total of 4,188 coding sequences (CDSs), 3 rRNAs, and 48 tRNAs were predicted.

The genome contained *pmoCAB* genes encoding particulate methane monooxygenase and *mmoRGXYBZDC* genes encoding soluble methane monooxygenase. The genome also contained *nifHDKENSU* genes for nitrogenase and its related functions, suggesting that strain 3S-1 potentially fixes atmospheric N_2_. Examination of N_2_ fixation and CH_4_ oxidation of strain 3S-1 could contribute to our understandings of the CH4-N cycle in the rice roots in paddy field ecosystems.

### Accession number(s).

This whole-genome shotgun project has been deposited at DDBJ/EMBL/GenBank under the accession no. LXWX00000000. The version described in this paper is the first version.
